# The Relationship of Severe Early Childhood Caries and Body Mass Index in a Group of 3- to 6-year-old Children in Shiraz

**Published:** 2014-06

**Authors:** A. Edalat, M. Abbaszadeh, M. Eesvandi, A. Heidari

**Affiliations:** a Dept. of Pediatric Dentistry, School of Dentistry, Shiraz University of Medical Sciences, Shiraz , Iran.; b Postgraduate Student or Pedodontist, Dept. of Pediatric Dentistry, School of Dentistry, Shiraz University of Medical Sciences, Shiraz, Iran.; c Dentist

**Keywords:** Preschool children, Body mass index, Dental caries, Software

## Abstract

**Statement of Problem: **Early childhood caries can cause pain, discomfort and also inability to have a healthy nutrition .Malnutrition can be characterized when there is a weight, height, and body mass index (BMI) deficiency.

**Purpose:** The aim of this study was to evaluate the relationship between the severe early childhood caries (based on the dmft index) and BMI in pre-school children in Shiraz.

**Materials and Method: **A descriptive analytical cross-sectional study was enrolled on 202 healthy preschool children with the age range of 3-6 years recruited from the kindergartens of different socio- economical parts of Shiraz, Iran. The Anthropometric measurements, weight and height were evaluated. The Z-scores were calculated employing WHO Anthro software (www.who.int/childgrowth/software/en/ index.html) to elucidate the subject’s status on the age- and sex-specific growth chart. Every Child who has received two Z-scores under the normal value (< -2) was considered as abnormal. The relationship between dmft index and BMI was then investigated.

**Results: **The mean of dmft was 4.13. From children with severe early childhood caries, 12.5%were under weight, 5% had height deficiency and 19.5% had BMI deficiency, however, there was no significant relationship between increasing dmft and the height, weight and BMI deficiency.

**Conclusion: **There was not a linear correlation between severe early childhood caries and BMI, height, and weight deficiency. An incidence of 55% was yielded for severe early childhood caries which was an additional finding of this study.

## Introduction

Oral health and overall health and well-being are inseparably related [1]. The role of nutrition in the maintenance of health, growth and also its relation to the dental caries is well known. Food choices and nutritional intake may affect the dental health. It might also be affected by poor dental health. Currently, there is a growing prevalence of early childhood caries in developing countries. This is mostly because of frequent use of junk foods, soft drinks, snacks, and inadequate dental hygiene [2-3]. Children would suffer from pain and malnutrition as a consequence of untreated dental caries, mostly attributable to the expensive dental treatments [4].

Caries of the primary teeth “Early Childhood Caries” (ECC) is one of the most prevalent health dilemmas in infants and toddlers [3]. It could be reflected as an epidemic problem in families with low income and in under developed countries [2-3]. ECC is deliberated as one of the major causes of hospitalization in young children, who essentially receive general anesthesia for extraction or restorative purposes [1-4].

Early childhood caries (ECC) is defined as the presence of one or more than one dental caries (with or without cavity), missing (caused by caries), or filled tooth surface in any primary tooth in a pre-school age child (71 months or younger) [3].

Severe early childhood caries (SECC) is a term defined as dmft score greater than 3 in children of 3-6 years old [5], or any "atypical", "progressive", "acute" or rampant forms of dental caries [6].

Growth is a significant indicator of child health; it is so crucial that the World Health Organization (WHO) recognizes it as the best single measure for delineating the nutritional condition and health of children. It is also conceded as an indicator to identify the quality of life in whole population [7].

The relationship between dental caries and body mass index (BMI) in children was evaluated in different countries and the results were inconsistent. In some advanced countries, frequent eating of food and high consumption of carbohydrates were reported to be the reason of increasing obesity and dental caries [8-9]. Conversely, in some developing countries dental caries resulted in malnutrition and inability of consuming food [6-7, 10].

The chronicity of childhood caries may have the same effect on child's ability to withstand normal growth patterns as any other chronic disease or infections, therefore, impact general health and well-being [7].

There are particular ways to follow up a child’s normal growth pattern. The growth charts consist of a series of percentile curves that illustrate the distribution of selected body measurements in children. The charts designated by CDC (center of disease control) and WHO growth charts are the examples [11].

WHO growth charts are considered as the standard charts. They characterize how children grow when optimal conditions are provided. However, clinicians often use CDC growth charts which identify how typical children in United States grow during a specific period of time [11].

WHO Anthro software (www.who.int/childgro-wth/software/en/index.html) is an application that facilitates the automatic analysis of individual data for children and permits specialists to measure and to compare human physical dissimilarities. The program features three modules: anthropometric calculator, individual assessment and nutritional survey. Moreover, this software employs the statistical software package (SPSS) to facilitate the analysis of study data [11].

In children and teenagers, with the age range of 2-20years, the amount of body fat change as the body grows and these alterations are different in boys and girls. These growth and gender- specific differences need to be taken into account in the BMI assessment for children and teenagers. These child- specific BMI values are referred to as “BMI for age” [9].

It is believed that dental caries may be deliberated as an imperative underlying factor for the condition of wrong dieting; it is able to influence child growth negatively. The current study also aimed to assess children with SECC to determine whether the SECC was associated with age-specific BMI, weight and height deficiency in a sample population of 3- to 6- year-old children. Many research investigated the relation between BMI and dental caries, however, this study used WHO Anthro software as a standard method of anthropometric and nutritional survey of children growth.

## Materials and Method

In this descriptive-analytical cross-sectional survey, a total of 202 children being 3 to 6 years old (101 boys and 101 girls) were recruited randomly from the kindergartens in different socioeconomic areas of Shiraz. All parents were informed thoroughly about the nature of the study and deliberately signed an informed consent. 

The data, regarding the height (centimeter); weight (kilogram), and dmft index were collected. The subjects were seated and the examination of the oral cavity and teeth were performed by direct observation, using the head light. Socio-demographic data of children including age, gender, number of family members, and parent’s education were recorded.

Medical history, number of dental visits, number of carious teeth, and the status of oral and dental hygiene were registered. Children with the past history of systemic disease were excluded from the study. The values height and weight of each child was transferred to WHO Anthro software version 3.2.2 [6]. Anthro soft-ware (www.who.int/childgrowth/software/en/index.html) was used to compare the data of our study population and the WHO standards for weight, and length/height for age. World health organization advocated this software to calculate the BMI and to recognize the status of health and fitness of children. Every child‘s score on the growth curves was traced. Then the curves of BMI, weight, height, and “height for weight” were scaled by z-score, calculated and traced. Since the Anthro software calculation differs for children aging below and above 60 month, the calculations for the two groups were performed separately. Each record with two standard deviations below the normal value (< -2), was esteemed as abnormal. Children were divided into two groups regarding their dmft index; more or less than 3. This number was adopted to distinguish the children with SECC. The curves of height-for-age Z-score (HAZ), weight-for-age Z-score (WAZ), BMI-for-age Z-score (BAZ) and weight-height z-score (WHZ) were traced. Then the relationship of dmft with all curves were verified and analyzed by fisher test.

## Results

From 202 participants, 80 children were above 60 month and the average age of the children was 57.9 months. The descriptive data is illustrated in Table 1.

**Table 1:** Descriptive data of sample population

	**Number of cases**	**MIN**	**MAX**	**Mean**	**SD**
Dmft	202	0	16	4.13	3.48
month	202	37.88	73.40	56.90	8.082

**Figure 1:**
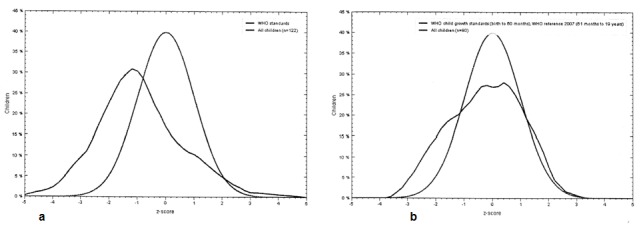
**a:**  Comparison of the height/weight proportion in the study population (under 60 month) with WHO standards.  **b:** Comparison of height/weight proportion in the sample population above 60 months with WHO standards.

Figure 1 displays the distribution of children, scaled by height/weight compared to the normal population. Regarding the BMI assessments, 39 (19.3 %) of patients had BMI deficiency, 25(12.3%) of them had SECC but there was not a statistically significant difference in dmft levels *(p*= 0.502) (Table 2).

**Table 2:** Comparison of the dmft level in the BMI deficiency group

	**BAZ groups**		**Total**
	**More than -2 Z-score**	**Equal/Less than -2 Z-score**	
dmft< 3	63	14	77
dmft ≥ 3	100	25	125
Total	163	39	202

Table 3 depicts the relationship of SECC and weight assessments, 25(12.3%) of patients had weight deficiency and 14(7%) showed SECC. There was no significant difference in dmft levels (*p*= 0.188).

**Table 3:** Comparison of the dmft level in the weight deficiency group

	**WAZ groups**		**Total**
	**More than -2 Z-score**	**Equal or less than -2 Z-score**	
dmft< 3	66	11	77
dmft≥3	111	14	125
Total	177	25	202

Table 4 shows the relationship of SECC and height deficiency; 10(4.9%) of patients had short stature for age, and only 6(2.9%) of them had SECC. No significant different was detected in dmft level of children with short height (*p*= 0.151).  In assessing the children for weight/height ratio; we found that 97 children were normal, 25 children implies the children were thin. However, no statistical significant difference was identified in their dmft levels. WHZ score was calculated in a total of 122 children who were under 60 months (Table 5).

**Table 4:** Comparison of the dmft level in the height deficiency group

	**HAZ groups**		**Total**
	**More than -2 Z-score**	**Equal or less than -2 Z-score**	
dmft< 3	73	4	77
dmft≥3	119	6	125
Total	192	10	202

**Table 5:** Comparison of the dmft level considering weight according to height

	**WHZ groups**		**Total**
	**More than -2 Z-score**	**Equal or less than -2 Z-score**	
dmft< 3	43	7	50
dmft ≥ 3	54	18	72
Total	97	25	122

## Discussion

Dietary habits have been enormously changed during the past decades in developing countries. The traditional “three main courses a day” has been replaced by frequent use of snacks, junk foods, soft drinks and so on. As a result, severe dental caries are happening in our society which seems to be an epidemic trait. Data from around the world explain that the reasons of most nutrition problems have not altered very much over the past 50 years. Poverty, unawareness and illness, together with insufficient food supplies, detrimental environments, social stress ,and prejudice, still carries on as a network of interrelating factors which merge to create circumstances in which malnutrition flourishes [10-11]. However, the approach to undertaking the malnutrition is altered significantly. Each decade perceives a new prevailing framework, pattern, solution or quick fix asserted to be competent of reducing the malnutrition problem before the decade have conceded [6, 8, 11]. Changing lifestyles and consumption prefigures have been a general aspect of most developing nations, in recent decades [11].

Our study found dmft as 4.13 that is in line with finding of study of Malek Mohammadi et al. (dmft=4.7) and Pourhashemi (4.44) enrolled in other regions of IRAN [7, 10].

Based on the study of Weddell and Klein, children had higher incidence of caries in low socioecononomic populations [12].

The relationship between frequent use of diet rich in carbohydrate, sucrose and ECC has been established. These diets which lack the proteins and micronutrients would probably have negative effects on the growth and development of children [4].

In Thomas and Primosch’s study [13], the weight of children who suffered from progressive caries was compared with the control group .They concluded that the children with ECC may not necessarily have low weight [13]. Perhaps, in their survey, using weight was not a proper indicator to assess the nutritional status which is characteristically a multifactorial premise.

In the current study, height-weight ratio was revealed, owing to the WHO charts presented by Anthro software in order to assess the healthy fitness. The outcome showed that 20.5% (25 out of 122) of children had deficiency in weight /height ratio, which means they were thin bestowing their height. 

Sheiham et al. [14] investigated the effects of dental caries on body weight, growth and quality of life in 3-6 year-old preschool children and concluded that children with severe childhood caries had less weight than the control group. Likewise, they reported that children had weight gain and improved quality of life after receiving the pertinent treatment [14].

Thomas and Primosch [13] conveyed that the parents of the studied children reported a significant improvement in children’s quality of life after receiving the appropriate treatment of dental caries.

One of the most accurate methods of BMI assessment in children is employing Anthro software designed by WHO, itemized for children of 3-5 years, both genders.

Pourhashemi and Golestan [10] reported that children with deficiency of "weight for age" had been significantly consuming soft drinks but there was not a linear correlation between dmft and soft drinks consumption [10]. According to our results, 12% of children had deficiency of weight, 7% of them had dmft above 3; which was considered as severe ECC. There was no significant difference in the dmft level of children with weight deficiency. Perhaps other factors such as pulp involvement or pain on chewing should be taken in to account to discriminate children with improper nutrition.

Pourhashemi and Golestan also found that children with insufficient height for age, categorized as “short stature” and weight for height, recognized as “being thin”, exhibited with high consumption of glucose and soft drinks [10].

In our study, 5% of children had deficiency of height which 3% of them was in SECC group, 19 % of children had deficiency of BMI which 12% of them had severe caries. There was not a significant different in the dmft level of children with low BMI.

The children with ECC, enrolled in the study of Slavkin and Baume [15], were underweight and have gained only 80 percent of the optimal weight which shows an aspect of failure-to-thrive child (FTT). FTT is an indicator of children who fail to show optimal growth throughout their life time [15].

A total of 12.3% of children were underweight in the current study. In a retrospective case study, Vania and Parisella [16] challenged the relationship between weight and ECC. They surveyed the body mass index in a group of ECC children (3 to 6 years old) and a comparable control group composed of caries-free children. They reported that, regarding the BMI percentile in children with ECC, 10% of the subjects were underweight, 55.9% had normal weight, 22.22% were at risk of overweight and 11.11% of cases exhibited with overweight. They realized that more children in the case group were underweight than in the control group and it was statistically significant (10% versus 4.94%). They concluded that the substantial number of underweight cases in SECC group might have been due to caries and missing teeth. This outcome was almost in line with our findings except that we were trying to find an association between SECC and growth deficiency elements [16].

The consequences of dental caries on weight deficiency occur immediately; however, the effect of malnutrition on the height of children takes a long time to appear. Therefore, even a little percentage of low height (4.9%) presented as Z-score, was imperative for us. 

Moreover, it would be better to consider parent’s BMI due to the close genetic relationship of child’s growth with this index.

It appears that reduction of BMI have the longterm impact on growth and development of individuals in early growth age [7].

To find a link between severe ECC and BMI or weight deficiency, the authors believe that the study should be done in larger number of population. Furthermore, the patients who have the pulp involvement, pain or difficulty on chewing should be individualized as a separate group. Undeniably, it is likely that not a single common risk factor plays a role in the relationship between ECC and BMI but rather a complex interface of health behaviours, social elements and genetic aspects that determines both caries and alterations in BMI. The child dental services and child health facilities can alter this relationship to positively improve the dental and nutritional health of children. Considering all the limitations of the study, our results provide additional evidence in the literature for the complexity of the relationship between ECC and BMI. 

## Conclusion

Under the limitation of this study, there was no significant relationship between increasing dental caries and decreasing height, weight, and BMI. However 55% of the children had severe early childhood caries which was an additional finding of this research. 
